# Prognostic biomarkers to identify patients destined to develop severe Crohn’s disease who may benefit from early biological therapy: protocol for a systematic review, meta-analysis and external validation

**DOI:** 10.1186/s13643-016-0383-5

**Published:** 2016-12-01

**Authors:** Steve Halligan, Darren Boone, Gauraang Bhatnagar, Tariq Ahmad, Stuart Bloom, Manuel Rodriguez-Justo, Stuart A. Taylor, Susan Mallett

**Affiliations:** 1 Centre for Medical Imaging, UCL, 3rd Floor East, 250 Euston Road, London, NW1 2PG UK; 2 Department of Radiology, Colchester Hospital University NHS Foundation Trust, Colchester General Hospital, Turner Road, Colchester, Essex CO4 5JL UK; 3 Department of Gastroenterology, Royal Devon and Exeter NHS Foundation Trust, Barrack Road, Exeter, EX2 5DW UK; 4 Department of Gastroenterology, University College Hospital, 235 Euston Road, London, NW1 2BU UK; 5 Department of Histopathology, University College Hospital, 235 Euston Road, London, NW1 2BU UK; 6 School of Health and Population Sciences, University of Birmingham, Edgbaston, Birmingham, B15 2TT UK

**Keywords:** Diagnostic accuracy, Review, systematic, Meta-analysis, Crohn’s disease, Biological markers, Biomarkers, Prediction, Prognosis

## Abstract

**Background:**

It is believed increasingly that patients with severe Crohn’s disease are best treated early with biological therapy, which may ameliorate subsequent disease course and diminish long-term complications. However, we cannot predict currently which new presentations of Crohn’s disease are destined to develop severe disease so treatment cannot be targeted to the most appropriate patients. Accordingly, via systematic review and meta-analysis we aim to identify if biomarkers of disease activity are able to predict development of severe disease.

**Methods/design:**

We will search the primary literature and conference proceedings for studies of biomarkers of all types including clinical, endoscopic, radiological, faecal, urinary, serological, genetic, and histological. Precise definition of “severe” disease is elusive so we will include sensitivity analysis to account for different definitions. We will use the CHARMS checklist to frame our question and to extract data. We will extract the study design, setting, participant characteristics, biomarker(s) investigated, and study outcomes. Bias will be assessed via the PROBAST tool. We will present the results using narrative and graphical methods. We will present the summary by meta-analysis where there are sufficient studies with reasonable homogeneity, using methods appropriate to the type of data extracted. Heterogeneity will be presented via Forest and ROC plots.

**Discussion:**

If this systematic review and meta-analysis identifies biomarkers that appear sufficiently predictive for subsequent severe disease course, we aim to combine them in a predictive model, followed by external validation using individual patient data. A predictive model able to identify new presentations of Crohn’s disease destined to develop severe disease subsequently would have considerable clinical utility for patient management.

**Systematic review registration:**

PROSPERO CRD42016029363.

**Electronic supplementary material:**

The online version of this article (doi:10.1186/s13643-016-0383-5) contains supplementary material, which is available to authorized users.

## Background

### Crohn’s disease and modern treatment strategy

Crohn’s disease is an inflammatory ulcerative enteropathy that tends to affect young adults and can be extremely debilitating. There is no cure, and treatment is traditionally applied in a “bottom-up” fashion, directed at symptoms when they arise and escalated when symptoms worsen. However, newer biological therapies appear to ameliorate ultimate disease trajectory, raising the possibility that early “top-down” treatment with these agents could “stop the disease in its tracks”. The first disease-modifying biological agent was infliximab, a monoclonal antibody against the cytokine TNF-α, binding with it and preventing receptor binding. A randomised trial of infliximab versus placebo found that of patients responding to an initial dose, half achieved complete mucosal healing after 1 year, stayed in remission longer, and discontinued steroids earlier than controls [1]. Biologicals also appear incrementally more effective when used in combination with other immunomodulators such as azathioprine [2], especially when administered in a “top-down” fashion [3, 4]. Newer agents such as adalimumab are also effective [5].

The REACT study randomised patients to conventional “bottom-up” therapy or “early combined immunosuppression”, finding major complications, hospitalisation and surgery reduced significantly at 24 months for intervention clusters [6]. Accordingly, current thinking is that early aggressive biological treatment combined with immunomodulation will prevent future disease and is preferable than merely responding to symptoms. However, administering biologicals early to all patients is unwise because these agents may precipitate serious infection, are hepatotoxic, and can cause demyelination, lupus syndrome and even lymphoma [7]. Biologicals are also very expensive. A strategy that could identify new diagnoses of Crohn’s disease destined to develop severe disease in the future would have considerable clinical utility by directing these patients to early biological treatment while avoiding this in others.

### Biomarkers of disease activity

Optimal therapeutic response can be defined by “deep remission”, a term that describes complete mucosal healing combined with Crohn’s disease activity index (CDAI) < 150, and which identifies patients who are responding to biological therapy where administered (although deep remission is not necessarily associated with severe disease in and of itself). Confident diagnosis of deep remission currently requires direct visualisation of the endoluminal bowel via endoscopy but the small bowel is most affected by Crohn’s disease (circa 75% of patients), while being relatively inaccessible to endoscopy; push enteroscopy is technically difficult and invasive, and capsule endoscopy is contraindicated in patients with bowel strictures, which are common in Crohn’s disease. A more acceptable “biomarker” acting as an effective surrogate for deep remission (and thus disease activity/response to therapy) would have great clinical utility.

According to the USA National Institute of Health, a biomarker is, “a characteristic that is objectively measured and evaluated as an indicator of normal biologic processes, pathogenic processes, or pharmacologic responses to a therapeutic intervention.” Biomarkers in Crohn’s disease may be able to indicate the presence or absence of disease and its severity. We do not wish to be too restrictive when labeling an intervention or characteristic as a “biomarker”. While the term is associated with novel diagnostic technologies, simple and effective biomarkers have been used for decades. For example, stool frequency reflects colonic inflammation directly and should not be excluded from systematic review because it is not “novel”. Smoking has a profound effect on disease outcome and should be included although smoking, in and of itself, is not a marker of disease activity. Several studies have investigated simple clinical factors predictive of an “aggressive” disease course and the Markov modelling of these has shown that disease activity over the year following diagnosis is predictive of clinical course over the following decade [8].

We therefore wish to identify the whole range of potential biomarkers used in Crohn’s disease, including clinical (both clinician and self-reported outcomes), endoscopic, radiological, faecal, urinary, serological (including the range from basic tests to antibodies), genetic, and histological. For example, C-reactive protein (CRP) is an acute-phase protein expressed by the liver that is used widely in clinical practice. Calprotectin, a protein released in an inflamed gut epithelium, is a more recent biomarker that has also reached daily practice. Calprotectin levels change with treatment. Lactoferrin is a similar protein biomarker. We anticipate that the diagnostic accuracy of such biomarkers may already have been subject to systematic review and meta-analysis. For example, one such review aimed to determine if calprotectin levels could differentiate between inflammatory and irritable bowel disease in children [9].

Because we anticipate that there will be many potential biomarkers, we will set quality/quantity thresholds for review inclusion that prevent us from extracting data for biomarkers that have been studied in insufficient numbers and/or with weak methodology (see the inclusion criteria below). For example, at the time of writing, more than 70 separate genes have been implicated in Crohn’s disease [10]. While genetic sequencing is presently very expensive and many individual genes have been studied in little depth, sequencing will become more cost-effective in the near future. Our systematic review must therefore consider those genes where sufficient primary studies exist. Genetic makeup is also linked to response to biological therapy. Since genetic makeup is fixed, these factors need only be measured once, as opposed to other biomarkers that fluctuate with disease activity. There are also multiple antibody candidates and prognostic strategies that have focused on both the titres of individual antibodies and the number of different antibodies. For example, patients with three or more positive antibodies are eight times more likely to need surgery than negative patients [11].

### The need for a systematic review

The United Kingdom National Institute for Health Research (NIHR) Health Technology Assessment (HTA) Programme has funded a systematic review and meta-analysis of the indexed medical literature to identify biomarkers that may be able to identify patients with Crohn’s disease who are destined to develop severe disease and who may therefore benefit from early biological therapy (see Acknowledgements). It is hoped that early identification of such patients will ameliorate the course of their future disease while avoiding overtreatment in others who do not need it or who will not respond. Achieving this necessitates the development of a prognostic model fed by data identified from systematic review.

### Results from a scoping review

We performed an initial “scoping review” in order to assess data likely available, both in terms of the individual biomarkers and the volume of studies associated with each. The scoping review was performed by a clinical researcher with content expertise in Crohn’s disease (GB), supervised by a senior member of the research team (SH) and was confined to 2013. Search terms and results are presented in Table 1. We focused our attention on the 35 clinical trials identified, and 13 appeared potentially eligible for a systematic review. We then investigated the following questions: Was it possible to extract data for Crohn’s disease severity (i.e. severe vs. non-severe); were new presentations reported, and could they be extracted separately; could a 2 × 2 results table be extracted for the biomarker(s) in question; was prognostic information provided?

**Table 1:** Results of scoping review to identify studies of biomarkers in Crohn’s disease, performed in March 2015

Search term	Number of articles identified	Number of clinical trials identified
Crohn’s disease	41,158	1962
Crohn’s	41,322	1867
All MeSH terms for Crohn’s disease	42,373	1992
All MeSH terms for biological markers	848,207	45,368
Crohn’s disease all MeSH terms and biological markers all MeSH terms	3308	225
Limit to 2013	338	35

As anticipated, there was little overlap in biomarkers investigated by individual studies. A wide range was studied that included vitamin D, granulocyte macrophage colony-stimulating factor (GM-CSF), DCE/DWI MRI, CDAI and CRP, serum calprotectin, faecal S100A12, nitric oxide, serum serotonin, NOD2insC, oxidative stress markers, thiopurine metabolites, anti-neutrophil cytoplasmic antibodies, and anti-saccharomyces cerevisiae mannan antibodies. Based on the scoping data, we anticipate that the number of potential biomarkers potentially available is large but proportionally few will be reported in detail sufficient for meaningful meta-analysis, that most studies will describe patients who relapse rather than new presentations, that specific identification of patients with severe disease will be difficult, that extracting 2 × 2 tables will only be possible from a minority of papers without contacting the authors and that existing models will be encountered rarely.

### Objectives

Our primary objective is as follows:

1. To perform four systematic reviews of the literature that cover separate biomarker areas, to assess biomarker-predictive ability for severe Crohn’s disease. Four reviews are necessary because we anticipate a wide range of biomarkers. The biomarker areas will be as follows: (1) serological and urinary, (2) clinical, imaging and endoscopic (including patient characteristics and symptoms), (3) genetic and (4) combinations of tests/biomarkers. Ultimately, we will summarise evidence across all four reviews thereby producing an overall synopsis (the protocol presented here is “generic”, intended to cover the four reviews and overview).

Our secondary objectives are as follows:

1. To compare predictors using direct and indirect comparison of study results. Direct comparisons between predictors from the same study constitute stronger evidence and will be preferred over indirect comparisons across different studies.
2. To explore heterogeneity among studies by analysing subgroups classified as specified in Additional file 1.
3. To conduct sensitivity analyses to examine our main assumptions and definitions as specified in Additional file 1. In addition, we will conduct sensitivity analysis based on studies with low or unclear risk of bias, i.e. excluding studies at a high risk of bias.
4. To develop and validate a prognostic model to identify patients destined to develop severe Crohn’s disease and who may therefore respond to early biological therapy. We will develop our own model using predefined predictor combinations identified via the prior systematic review. We will externally validate our model via individual patient data (IPD). We will also examine and validate any existing models identified via systematic review.

The ability to examine primary and secondary objectives will be highly dependent on the availability and quality of data from published studies.

## Methods/design

### Ethical approvals

Ethical permission is not required by our institution for systematic reviews of available medical literature. However, the validation phases of the proposed research will require IPD. In the first instance, we anticipate IPD being drawn from the METRIC [12] and PANTS trials (http://public.ukcrn.org.uk/search/StudyDetail.aspx?StudyID=14175), to which we have IPD access. The ethical permissions necessary to access and use these data for the purpose of developing and validating a prognostic model will be sought. Should we fail to achieve the ethical approval, then this aspect of the study will not proceed.

This protocol has been drafted in line with the PRISMA-P checklist (Additional file 2).

### Eligibility criteria for inclusion in the review

- Primary studies will report patients with a proven diagnosis of Crohn’s disease in whom a biomarker(s) is used to predict the development of a severe disease (or vice versa).
- We will apply no age restriction but will extract paediatric subsets where these are reported (defined as age less than 16 years).
- Both new and established diagnoses of Crohn’s disease will be eligible because, while our focus is prediction of patients destined to develop severe disease (which implies that primary studies include patients with a new diagnosis), we anticipate that the large majority of studies will investigate patients with an established disease since these are far more numerous and accessible to researchers. Where possible, we will extract information relating to new and established subsets separately.
- Studies reporting all severities of Crohn’s disease will be eligible. Where available, we will extract information relating separately to subsets of patients with “severe” and “non-severe” disease (see explanatory paragraph below).
- Individual biomarkers will be reported in at least five individual primary studies.
- Not more than five individual biomarkers identified as “promising” by an expert panel but reported in less than five individual primary studies will be included in each of the four systematic reviews that we intend to conduct.
- Any univariable or multivariable models identified that report predictors of severe disease for patients with proven Crohn’s disease. In the case of models, we will extract data irrespective of the number of models that report an individual predictor or combination of predictors.
- We will apply no language restriction (we will arrange for translation for potentially important non-English research although we anticipate this will be a small proportion).

A precise definition of “severe” disease is elusive. The Montreal classification (a modification of the Vienna classification) is a phenotypic classification based on age at diagnosis, disease location, and disease behavior; stricturing (B2) and penetrating (B3) disease (together, 20% of patients) comprise those with severe disease [13]. The term “disabling Crohn’s disease” was introduced in 2006 [14] and includes patients presenting under 40 years of age, steroid dependency, hospitalisation, persistent symptoms for more than 1 year in a five-year period, extra-intestinal complications (notably perianal disease), a need for surgery, and a need for immunosuppression. The UK National Institute for Healthcare and Clinical Excellence technology appraisal guidance 187 of May 2010 titled “Infliximab (review) and adalimumab for the treatment of Crohn’s disease”, defined severe disease as “very poor general health and one or more symptoms from weight loss, fever, severe abdominal pain and usually frequent (3–4 or more) diarrhoeal stools daily[15]. People with severe active Crohn’s disease may or may not develop new fistulae or have extra-intestinal manifestations of the disease”. The NICE guidance goes on to state that “This clinical definition normally, but not exclusively, corresponds to a Crohn’s disease activity index (CDAI) score of 300 or more, or a Harvey-Bradshaw score of 8 to 9 or above.”

We will therefore not stipulate a single definition of “severe” disease for primary studies since we believe this would result in excessive discarded data, but will include sensitivity analysis for the different definitions of severe disease encountered. We will also consult our investigator group and collaborators so as to arrive at a robust and generally accepted definition of “severe” for the purposes of this review once we are aware of the range of definitions presented in the extracted data.

It should also be noted that our remit is to distinguish patients with Crohn’s whose disease is destined to become severe from those whose disease is not destined to become severe. Studies that employ controls that do not have Crohn’s disease (e.g. normal volunteers) may identify factors that are significantly associated with Crohn’s disease, but it is important to appreciate that such factors may not be associated with “severe” disease. Such studies will be included in the review and data relating to disease onset, severity and biomarker(s) extracted as described elsewhere. It is also possible that there will be biomarkers that identify non-severe disease, the absence of which could identify patients with severe disease.

In advance of the study identification and extraction, we will convene our investigator group to discuss a priori criteria that define whether an individual biomarker has been researched in enough depth to present a reasonable chance that primary studies will be sufficient to permit an accurate reflection of diagnostic accuracy via meta-analysis. A simple metric will be required, likely related to the individual number of primary studies identified for a specific biomarker in combination with a minimum number of patients studied by each. Our group will also define the date range over which primary studies will be identified; at the time of writing, we anticipate this will be from 1980 to the present day.

### Search strategy

We will use resources that enable us to search multiple databases simultaneously, from 1980 until the present day: the biomedicine subset of UCL MetaLib searches AHMED, BioMed Central, CINAHL plus, Cochrane, EMBASE, OVID, Pubmed and SCOPUS. We will report our search string as an Appendix to published studies. We will handsearch conference proceedings (European Crohn’s and Colitis Organisation, United European Gastroenterology Week, Digestive Disease Week) from 2012 to date inclusive in order to identify grey literature. A draft for the search strategy to be used for the PUBMED online database is reproduced in Additional file 3.

We will identify predictors recommended or mentioned in clinical guidelines or recommendations from established clinical associations (e.g. European Crohn’s and Colitis Organisation, ECCO; European Society of Gastrointestinal and Abdominal Radiology, ESGAR). Via our expert panel, we will identify prespecified predictors that are already in widespread clinical use. So as to not miss new predictors, our expert panel will also identify recent “promising” markers from abstracted data presented at relevant subspecialty meetings during the 2 years prior to the review, which have not yet appeared in sufficient indexed articles to meet our inclusion criteria. In order to avoid being swamped by large numbers of abstracted biomarkers studied in insufficient depth, we will limit the number selected to no more than five “promising” predictors for each individual review.

### Data collection

We will follow the CHARMS checklist for framing our systematic review question and to extract data [16]. To reduce costs, a single clinical researcher (DB) with content expertise in Crohn’s disease will perform the bulk of the extraction. In order to ensure that extraction proceeds correctly and in an unbiased fashion, we will pilot data extraction on a subset of 20 papers extracted by both the researcher and the senior members of the team (SH, SM). This procedure will assess both adequacy of the extraction sheet to capture the data necessary and also provide an opportunity to assess an inter-observer agreement. If disagreement is <5% (which we anticipate for these type of data following the scoping review described above) we will proceed with a single researcher. If we identify a particular item as problematic, a second researcher will also review this item. From our prior experience, we anticipate any difficulties will most likely relate to extraction of 2 × 2 tables and other numerical results, and so the second researcher will likely be a statistician. The researcher(s) performing the extraction will have easy access to senior members of the research team when questions arise regarding primary study suitability both for inclusion and/or the precise nature of the data extracted (methodology experts SM, SH and disease experts TA, SB).

Following piloting, DB will screen the titles and abstracts of all primary studies identified by the search string and determine whether these meet the inclusion criteria. Data will be extracted into the study extraction sheet developed specifically for the review; development will occur at a series of face-to-face meetings of the core research team. Where necessary, the statistician will help with the extraction of data for meta-analysis. Additional data will be sought from authors of primary studies where appropriate.

### Data items to be extracted

The extraction sheet will include the following items as a minimum:

- Details of study design (e.g. cohort, randomised controlled trial, retrospective database, routinely collected data) and study methods.
- Setting/context (organisation/service type, country).
- Participants, including age and range, gender, whether the diagnosis is new or established (symptom duration and/or time since diagnosis for established disease), symptom severity (and how this is defined), disease location and burden, disease complications, HBI, CDAI etc. (where these are not the primary biomarker under investigation), details of any surgery, anal disease and continence outcomes. As noted previously, we expect the exact definitions of severe disease and disease remission to vary between studies so we will note specific definitions and include sensitivity analyses for definitions of outcomes.
- Biomarker(s) used/investigated (including pre-analytical methods and analytic measurement methods, frequency of measurement), adverse events related to biomarker administration, reliability and reproducibility of biomarker measurements.Where available, we will collect information on unit costs of biomarkers.
- Where biomarker measurement could result in adverse effects, we will collect relevant information to summarise these data. We will highlight issues and information where available on the reliability and reproducibility of biomarker measurements, including how this may affect reliability of predictions using these biomarkers.
- Study interventions and outcomes (including definitions, thresholds for severity/remission and whether prespecified), median follow-up time with interquartile range and range (we will conduct sensitivity analyses for different time intervals).
- We will consult both our patient and public involvement (PPI) representative and METRIC/PANTS expert panels to identify other important outcomes.

Where models are encountered, we will extract the type of model study (development, internal validation or external validation), included predictors (including methods of measurement, categorisation of continuous outcomes, blinding to outcome assessment and predictor variables), sample size (number of participants with events and included in modelling), statistical modelling methods where present (including model fitting, treatment of missing data, methods used to adjust for overfitting), model performance (discrimination, calibration, sensitivity, specificity, net benefit, reclassification), model estimates and 95% confidence intervals (e.g. unvariable unadjusted or adjusted estimates for predictors, adjusted coefficients for predictors in multivariable models). We anticipate that data may include estimates with 95% CI including odds ratios (OR), risk ratios (RR), hazard ratios (HR), survival curves and log rank estimates for time-to-event models, sensitivity, specificity, positive predictive value, and negative predictive value. Where possible we will extract 2 × 2 tables underlying the data using excel conversion spreadsheets: For data expressed using sensitivity/specificity/NPV/PPV, we will use methods developed by Deeks and Snell (Deeks and Snell, personal communication), and for data expressed as hazard ratios or survival curves we will use methods based on Tierney [17] and Parmar [18]. Unadjusted estimates will be preferred, with adjusted estimates only where unadjusted are unavailable.

### Assessment of risk of bias in individual studies

We will use the PROBAST (Prediction study Risk Of Bias ASsessment Tool) to assess the risk of bias in prediction modelling studies using a prepublication version with permission of the PROBAST Steering group [19]. The tool has five broad domains: patient selection, index test, reference test, flow and timing and analysis. At the present stage, we intend to omit the fifth domain for assessment of single predictors from univariable analyses.

### Summary measures and results synthesis

We will present results using narrative and graphical methods, where study results are obviously heterogeneous by visual inspection, or where results from different studies are presented using statistical measures that we cannot combine, or for multivariable prediction models with few studies (where we will extract data even if there are fewer than five studies).

We will use the following methods where there are sufficient studies allowing extraction of results in the same format with reasonable homogeneity to allow summary by meta-analysis:

- For time-to-event data, we will use random effects inverse variance meta-analysis methods (DerSimonian and Laird) where hazard ratios and standard errors can be extracted [20].
- For odds ratios extracted as 2 × 2 tables, we will use stratified one-stage random effects models, ensuring correct clustering of patients within studies by using separate intercepts for each study [21, 22]. The binary one-step approach using the exact binomial distribution is preferred over other meta-analysis methods (DerSimonian and Laird, Mantel Haenszel, Peto’s Odd ratio); as in these data, the event rate is low with many zero cells (requiring continuity correction when other methods are used) and comparison arms (number of patients with/without biomarker of interest) are highly unequal [21–25]. Univariable meta-analysis of outcomes will be completed where there are more than three studies for each outcome, biomarker or biomarker subgroup. For odds ratios reported as coefficients and standard errors only, we will use random effects inverse variance effects (DerSimonian and Laird) [20].
- For data that can be extracted as 2 × 2 tables as sensitivity and specificity, we will use a bivariate meta-analysis [26] using the “xtmelogit” command (STATA 14, StataCorp LP, Texas, USA).
- Where 2 × 2 tables can be extracted for biomarkers at different thresholds, we will present the results using SROC and where there are sufficient studies including hierarchical meta-analysis [27]. Results will be presented for sensitivity values at a fixed specificity value, based on clinical consensus regarding the relative potential consequences of over and under diagnosis, i.e. misclassification costs [28].
- Where IPD data are available, multivariable models will be fitted to data where more than one biomarker is included per patient, enabling analysis of potential confounding between the biomarker and other predictors.
- Where appropriate, we will use a mixed multilevel subject-specific (conditional) analysis, fitted by adaptive Gaussian quadrature using ten integration points or two if required for model convergence (using the “xtmelogit” command (STATA 14, StataCorp LP, Texas, USA).
- Where models do not converge because of inability to estimate all parameters, we will (i) conduct separate univariable meta-analysis instead of bivariate meta-analysis for sensitivity and specificity and (ii) for data on rare events, results will be pooled as if from a single study for odds ratios. For meta-analysis of bivariate outcomes (sensitivity and specificity), where specificity values are 100%, we will undertake a univariable meta-analysis of sensitivity and calculate the exact 95% confidence interval for the 100% specificity estimate using the total number without disease across all studies as the denominator [19].

We expect heterogeneity in study estimates due to variability in outcome definitions, in patient populations, biomarker test methods, methods for developing models, and confounding factors. Heterogeneity is often informative and will be presented via forest plots, ROC plots, and where there are sufficient studies the presence of heterogeneity will be tested within meta-analysis. Our model output will be subject to uncertainty related to the input variables and we will attempt to quantify this via sensitivity analysis. Planned sensitivity analyses at this stage are as follows: for definitions of outcomes (since exact definitions and/or scales of severe disease and disease remission are likely to vary between studies), for different time intervals since diagnosis (since studies may not be divided simply into those with new and/or established diagnoses, and definitions of the duration of established disease will vary by study) and restricted to studies with low or uncertain bias (i.e. excluding those with a high likelihood of bias). Planned sensitivity analyses are detailed in Additional file 1.

### Systematic review registration

This systematic review is registered with PROSPERO: CRD42016029363.

### Model development and validation

Where existing models or biomarkers are identified that can be externally validated using our own IPD, we will examine individual predictors and predictors in combination, both using predictor weightings from identified models and using predictor weightings from our own models developed using predefined predictor combinations. We will express results in terms of calibration, discrimination, sensitivity at a fixed specificity identified by our panel as clinically relevant [29]. We may update these models by recalibration where applicable. We will seek further IPD datasets and information from authors where additional details are needed to allow validation.

Ultimately, we wish to provide an overall synthesis of evidence from the systematic review, univariable analysis, and any models developed and validated via IPD. We will interpret clinical utility in conjunction with our expert panels and provide recommendations and guidance. Finally, we will propose a model and trial design to tune/test this in a subsequent larger prospective external validation.

### Patient and public involvement (PPI)

We have included a patient as a collaborator on this research so as to facilitate patient and public involvement. He will facilitate access to patients and their representative groups and will help the investigators maintain a patient-centric focus to the proposed research.

## Discussion

At the time of writing, there are many narrative reviews that describe a large variety of individual biomarkers potentially applicable to Crohn’s disease, the research data arising from their investigation, and their potential application in clinical practice. However, it is our experience that none of these reviews assemble the totality of available information regarding biomarkers (much of which is contradictory) into a format that clinicians can use to guide their day-to-day management of individual patients. For that reason, clinicians need urgently a systematic review that summarises the current literature. Clinicians also seek a model that combines values from disparate biomarkers to provide a unified and comprehensible metric that describes the overall picture of prognosis and disease trajectory in an individual patient. This information could then be used to guide the therapeutic decision whether or not to administer early biological therapy when balanced against the risks and costs of prescribing. Therefore, this systematic review will identify potential biomarkers and models that may have utility to identify those patients destined to develop severe Crohn’s disease in the future. We will identify the most promising predictors and, where possible, test them using IPD in the context of a prognostic model.

While we do not anticipate identifying a substantial number of models, if any, it is possible that existing predictive models of biomarkers exist. If so, these will need validation using IPD and possible incorporation into our own model if found sufficiently predictive.

The clinical utility of predictive biomarkers is hindered greatly by the fact that evidence levels for individual biomarkers varies widely and many have not been studied with sufficient methodological rigor to recommend clinical application. For example, while increased levels of a biomarker (e.g. in blood and stool) may occur in patients with Crohn’s disease, evidence of how this can be used to predict subsequent patient outcomes is usually weak. Our preparatory examination of the available literature suggests the best existing evidence is available for CRP and calprotectin. An evidence-based review of current biomarker models, biomarkers suitable for inclusion in models, prioritisation of model(s) for external validation, and external validation of models with IPD biomarkers would have considerable clinical utility.

It is important to understand that response to biological therapy is not universal, and that not all patients destined to develop severe disease will necessarily benefit from their early administration. It is our belief that a systematic review to identify biomarkers associated with response to biological therapy could be useful in that these biomarkers could be integrated into an overall model that considered both predictions of disease severity and therapeutic response. This may form the basis of future work should the current model be found sufficiently predictive.

## Additional files

Additional file 1: Crohn’s systematic review definitions and analysis. (DOC 99 kb)
Additional file 2: PRISMA-P 2015 Checklist. (DOCX 31 kb)
Additional file 3: Draft search strategy to be used for the PUBMED online database. (DOCX 104 kb)
