# Supplementary material for: Prognostic biomarkers to identify patients destined to develop severe Crohn’s disease who may benefit from early biological therapy: protocol for a systematic review, meta-analysis and external validation
Source: Syst Rev. 2016 Dec 1;5:206. doi: 10.1186/s13643-016-0383-5 (PMC5131410; doi:10.1186/s13643-016-0383-5)
Supplement: Additional file 1: — Crohn’s systematic review definitions and analysis. (DOC 99 kb) [file 13643_2016_383_MOESM1_ESM.doc]

# Additional file 1: Crohn’s systematic review definitions and analysis

| **SUMMARY OF REVIEW**  **TITLE:** Prognostic biomarkers to identify patients destined to develop severe Crohn’s disease.  **PRIMARY OBJECTIVE:** To assess the predictive ability of biomarkers for severe Crohn’s disease.  **SECONDARY OBJECTIVES: (**1) To compare predictors using direct and indirect comparison of study results. (2) To explore heterogeneity as defined below (3) To explore sensitivity as below.  **EXPECTED CHANGES OVER TIME DUE TO DIAGNOSIS OR TREATMENT DIFFERENCES:** In forest plots, studies will be ordered by publication year, to see any TNF effect over time. It is not possible to split studies into a pre- or post-TNF treatment era, as this treatment was introduced at different times across countries. | | | | | |
| --- | --- | --- | --- | --- | --- |
| **PARTICIPANTS**: new and established diagnoses of Crohn’s disease | | | | | |
| **Reason for potential groupings or categories. Give categories.** | **Report which categories will be separate or combined.** | **Data extraction: Report any priority order for categories** | **Presentation and MA: How are categories included** | **Is sensitivity analysis planned by category? Give details** | **Is heterogeneity analysis planned by category? Give details.** |
| **Diagnosis (2 categories)**   1. Newly diagnosed (typically within 3 months, but will include up to 6 months) 2. Patients with ongoing Crohn’s | Categories kept separate | Not applicable | Forest, SROC and MA^2^ separately by diagnosis | No | New vs ongoing^1^ |
| **Participant age (2 categories)**  Paediatric Crohn’s is seen as a distinct disease from adult Crohn’s.   1. paediatric Crohn’s (less than 5 years) 2. non-paediatric Crohn’s (>6 years) | Categories kept separate | **Paediatric group**  Where a study reports results for multiple age ranges, these will be combined where possible. If they cannot be combined, then one result will be used per study, based on largest number of participants in the age group^3^. | Forest, SROC and MA^2^ separately by paediatric and non-paediatric  For non-paediatric Crohn’s  See age as a baseline predictor for further sub categories of age | No | Paediatric vs non paediatric^1^ |
| **Unit of analysis:** Per participant  Single category only | Not applicable | Not applicable | Not applicable | Not applicable | Not applicable |
| **TARGET CONDITION**: Severe Crohn’s disease, disabling Crohn’s disease  **OUTCOME EVENT DEFINITIONS INCLUDED**: Beaugerie, Liege, modified Beaugerie, NICE, Paris, first surgery, relapse, time to first TNF therapy, other  **Variation in reference tests**: Different outcome types, different scales, different thresholds, different time of outcome assessment | | | | | |
| **Reason for potential groupings or categories. Give categories.** | **Report which categories will be separate or combined.** | **Data extraction: Report any priority order for categories** | **Presentation and MA: How are categories included** | **Is sensitivity analysis planned by category? Give details** | **Is heterogeneity analysis planned by category? Give details.** |
| **Outcome types**  Most studies are reporting by outcomes of   1. Severe or disabling Crohn’s 2. Relapse (may overlap to other outcomes) 3. First surgery 4. Hospitalisation 5. Time to first TNF therapy | Outcome groups can overlap e.g. a relapse may be a relapse of corresponding to severe Crohn’s, or it may require hospitalisation.  Outcomes will be presented separately, but also may be combined where outcomes based on similar components. | All reported outcomes to be extracted | Forest, SROC and MA^2^ by (i) outcome type (ii) by grouped outcomes based on common components^4^ | All in similar components in target definition vs subset of pre-defined (excluding newly developed severe disease definition) | Where outcomes have been grouped by common components, heterogeneity analysis for this grouping by separate outcomes^4^ |
| **Scale used to measure severe Crohn’s or relapse outcome**  There are several common distinct definitions based on different reference tests e.g. Beaugerie, Liege, modified Beaugerie, NICE, Vienna, stricturing or penetrating disease (Paris/Montreal), CDAI, other | Some papers will have their own definition of severe disease. We will record these with the components used e.g. surgery + steroids, so these can be aligned with closest standard definitions. | Where there is more than one definition of severe disease per publication (more than one scale or more than one cut point on a scale), we will extract up to 3 definitions of severe disease per study. We will give preference to extraction of definitions or thresholds based on pre-published and/or pre-specified definitions of target condition^3^. | Forest and SROC by closest scale for severe disease  MA^2^ across all definitions of severe disease, and where sufficient studies by outcome scale^2^. | All vs common definitions clearly pre-defined^2,4^ (i.e. excluding definitions of severe disease not pre-defined but developed within a publication) | Forest, SROC and MA^2^   1. By type of severe Crohn’s (e.g. Beaugerie) 2. By components in severe Crohn’s definitions^4^ 3. By separate outcome scales^4^ where outcomes grouped by common components |
| **Thresholds for severe Crohn’s disease or relapse scales**  Different thresholds can be used within a disease severity scale to define severe or disabling disease on a scale e.g.  CDAI >300 | Common thresholds will be identified and studies grouped within 10% of common values (Where highest scale value is 450, an error of 22 on either side of any scale value will be grouped with the common threshold) | Where a study reports more than one threshold for definition of severe disease, up to three thresholds will be reported. Commonly used thresholds will be preferentially extracted^3^. | Forest, SROC and MA^2^ by (i) common thresholds within a scale (ii) any definition of severe^4^ | MA^2,4^ of exact common threshold vs all studies within 10% of a common threshold^4^ | Forest, SROC and MA^2,4^ by different common thresholds |
| **Time of outcome assessment**  Time of follow up   1. Up to 12 months 2. 13-24months 3. 3-5 years 4. >5 years   Note studies less than one month excluded. | Categories will be reported separately.  Categories for 3 or more years may be combined where there are few studies.  As a secondary analysis, follow up categories will be combined to (i) 24 months or less (ii) >24 months | Where time ranges are reported differently in studies, we will use closest category for likely time of event for majority of participants in study e.g. 0-18 months would be 12months if events likely after 12 months. If not possible to establish from dataset, we will use literature sources to understand likely clinical context when events most likely.  Extract typically 3 time points per study (prioritise earlier time ranges) ^3^ | Forest, SROC and MA^2,4^ by closest to common time points  Forest, SROC and MA^2,4^ across all time points | MA^2,4^ by exact time vs closest to common time | Forest, SROC and MA^2^ by time of follow up |
| **Outcome measures**  **Types of outcome measure**   - 2x2 table - OR - RR - HR - Sensitivity and specificity - AUC (c-index)   **Variations on outcome measures**   - Unadjusted^5^ - Adjusted^6^ | 2x2 tables will be extracted for MA of OR^9^. OR and RR reported where possible will be converted to 2x2 tables.  Sensitivity and specificity with associated thresholds will be converted to 2x2 tables where possible for MA of OR^10^.  HR will be used for MA^7^. If only survival curves are presented these will be converted to HR^8^. | Unadjusted estimates will be preferred, but adjusted estimates (with adjustment variables) will be extracted if unadjusted not available^3^ | Forest and MA^2^ by outcome measure. | Not applicable | Not applicable |
| **PREDICTORS:** Clinical, endoscopic, serological (simple, genetic, abs), histological, stool tests -fecal calprotectin, imaging, urinary.  **GROUPING OF PREDICTORS IN REVIEWS:** Predictors will be grouped into four separate reviews (1) serological and urinary biomarkers (2)clinical, imaging and endoscopy where clinical includes patient characteristics and symptoms (3) genetics (4) combination tests including biomarker or genetic tests.  **RESTRICTING REVIEW TO MOST RELEVANT PREDICTORS**: To focus on predictors with potential clinical relevance, we will only include predictors where:   - Predictors are already recommended or mentioned in clinical guidelines or recommendations from clinical association (e.g. ESGAR, Royal College). - Pre-specified predictors that are already in clinical use. - Individual predictors with 5 or more studies included in the systematic review. - Recent “promising” markers with fewer than 5 studies identified by abstracted data. Not more than five “promising” predictors will be chosen by our expert panel per individual review (rationale: not to miss new promising predictors balanced against volume of data for new biomarkers). | | | | | |
| **Reason for potential groupings or categories. Give categories.** | **Report which categories will be separate or combined.** | **Data extraction: Report any priority order for categories** | **Presentation and MA: How are categories included** | **Is sensitivity analysis planned by category? Give details** | **Is heterogeneity analysis planned by category? Give details.** |
| **Underlying predictor**  **Genetic variants** e.g. NOD2 different genotypes (alleles) and their consequences. We will group by gene regardless of variant or method used to measure gene. We will also group gene and peptide variations linked to a gene.  **Biomarkers:** group by biomarker regardless of method of analysis. | Related components of an underlying predictor will be combined, but with details of variation in methods and thresholds recorded.  Different unrelated genes, biomarkers or clinical components will be kept separate. | Only tests where there are >5 included studies will be extracted. | Forest, SROC and MA^2^ by test. | No | Forest, SROC and MA^2,4^ by different test methods. |
| **Different thresholds** used to define a positive result | Common thresholds will be identified and studies grouped within 10% of common threshold values (Where highest scale value is 450, an error of 22 on either side of any scale value will be grouped with the common threshold) | We will present results at typical thresholds for a test.  We will prioritise extraction of results at thresholds used in clinical guidelines, manufacturer instructions, or published papers.  We will typically extract a maximum of 3 thresholds for each test^3^. | Forest, SROC and MA^2^ by grouping of close to common thresholds within a test | MA^2^ by exact common threshold vs all studies within 10% of a common threshold | Forest, SROC and MA^2,4^ by different common thresholds |
| **Disease severity**  Disease scores e.g. Vienna/Paris are sometimes used to stratify at baseline.  Category could be by severity of disease or type of disease (e.g. stricturing or penetrating) |  | Categories will be extracted using commonly used categorisation of disease severity where possible, or otherwise according to author groupings. Author groupings will be assigned to closest common definitions where possible. | Forest, SROC and MA^2^ by common category assignment. | MA^2,4^ by exact definitions of outcome vs nearest common category | Forest, SROC and MA^2,4^ by common category assignment |
| **Participant age for non-paediatric Crohn’s**  Previous work has proposed relationship to age. For non-paediatric Crohn’s we will use results from age categories   1. child, typically 5 to 18 years 2. young adult typically 18 to 39 years, 3. adult older than 40 years 4. all ages | Categories will be kept as separate age categories. In addition the age categories will be combined within a study. | If a study reports results separately from more than one age range within an age category, either results will be combined or only one result from an age category will be extracted per study.  The age range closest to the median age of the pre-specified categories will be preferentially extracted.  If a study reports results separately from more than one age category, these will be extracted.  Where it is not possible to separate results by age, the all ages category will be used. | Categories will be meta-analysed for separate age categories^1^.  Categories will be combined within a study where possible to obtain a separate analysis across all ages. This will be presented with a median or average age within the study  If a study uses different age categories results will be grouped to the closest age category for the majority of those with an event. | MA^2,4^ by age less than 40 years old vs all | Forest, SROC and MA^2,4^ by pre-specified age categories |

**Footnotes**

^1^Only possible where there are sufficient studies i.e. four or more studies in a group.

^2^MA will only be done if (i) there are four or more studies where the same outcomes measure can be calculated from the study (ii) study results are sufficiently homogeneous visualised in Forest or ROC space for a meaningful representation by a single summary statistic.

^3^Priority order of data extraction means that not all data is extracted from published articles.

^4^To avoid over representing results from a study in meta-analysis results, we will only include only one set of results per predictor within a category from each study.

^5^ Unadjusted measures are univariable analysis

# ^6^ Adjusted measures result from multivariable analysis where the prediction results relate to a combination of predictors, the prediction factor itself and the components included in the adjustment.

# ^7^ DerSimonian R, Laird N. 1986 Control Clin Trials.7(3):177-88. Meta-analysis in clinical trials.

^8^ Parmar MK, Torri V, Stewart L. Extracting summary statistics to perform meta-analyses of the published literature for survival endpoints. Statistics in medicine. 1998;17(24):2815-34. PubMed PMID: 9921604.

^9^ Debray TP, Moons KG, Abo-Zaid GM, Koffijberg H, Riley RD. Individual participant data meta-analysis for a binary outcome: one-stage or two-stage? PLoS One. 2013;8(4):e60650

^10^ Bivariate analysis of sensitivity and specificity produces informative summary measures in diagnostic reviews. Reitsma JB, Glas AS, Rutjes AW, Scholten RJ, Bossuyt PM, Zwinderman AH. J Clin Epidemiol. 2005 Oct;58(10):982-90
