# Supplementary material for: Prognostic biomarkers to identify patients destined to develop severe Crohn’s disease who may benefit from early biological therapy: protocol for a systematic review, meta-analysis and external validation
Source: Syst Rev. 2016 Dec 1;5:206. doi: 10.1186/s13643-016-0383-5 (PMC5131410; doi:10.1186/s13643-016-0383-5)
Supplement: Additional file 3: — Draft search strategy to be used for the PUBMED online database. (DOCX 104 kb) [file 13643_2016_383_MOESM3_ESM.docx]

**Additional file 3:**

Draft search strategy to be used for the PUBMED online database:

((crohn*) AND (Aggressiv* OR Sever* OR Disabling OR Montreal OR Beaugerie OR Liege OR Flare OR Penetrat* OR Strictur* OR Resection OR Surgical OR Surgery OR Stoma OR Failure OR Active OR Adverse OR Harvey-Bradshaw OR HBI OR CDAI OR index OR Perianal OR Complex) AND (Biomark* OR Marker OR Assay OR Imaging OR Radiolog* OR Genetic OR Examination OR Serum OR Blood OR Serolog* OR Stool OR Faecal OR fecal OR feces OR faeces OR Frequency OR Urin* OR Endoscop* OR histolog* OR histopathol* OR antibod* OR age OR Smoking OR test) AND (course OR prognos* OR outcome OR cohort OR progres* OR Predict* OR Risk* OR Outcome OR onset OR Biomarker* OR Natural history OR Predict*[tiab] OR Predictive value of tests[mh] OR Scor*[tiab] OR Observ*[tiab] OR Observer variation[mh] OR risk prediction model[tiab] OR predictive model[tiab] OR predictive equation[tiab] OR prediction model[tiab] OR risk calculator[tiab] OR prediction rule[tiab] OR risk model[tiab] OR statistical model[tiab] OR cox model[tiab] OR multivariable[tiab] OR validate OR nomogram OR predictive model OR validation OR prognostic model OR prognostic scor* OR prognostic index OR predictor OR diagnos*)) NOT ((review[Publication Type] OR Bibliography[Publication Type] OR Editorial[Publication Type] OR Letter[Publication Type] OR News[Publication Type])) AND ("0001/01/01"[PDat] : "2016/01/01"[PDat]) AND Humans[Mesh] )
